# Supplementary material for: Microbiota assembly, structure, and dynamics among Tsimane horticulturalists of the Bolivian Amazon
Source: Nat Commun. 2020 Jul 29;11:3772. doi: 10.1038/s41467-020-17541-6 (PMC7391733; doi:10.1038/s41467-020-17541-6)
Supplement: Supplementary file 1 — Supplementary Information [file 41467_2020_17541_MOESM1_ESM.pdf]

Sprockett *et al.,* "Microbiota Assembly, Structure, and Dynamics Among Tsimane Horticulturalists of the Bolivian Amazon"

Supplementary Information

Supplementary Figure 1. Dominant bacterial families of Tsimane stool microbiotas.
Supplementary Figure 2. Dominant bacterial families of Tsimane oral microbiotas.
Supplementary Figure 3. Gut and oral microbiota sharing between infants and mothers or unrelated adults.
Supplementary Figure 4. Gut and oral microbiotas diverge as infants age.
Supplementary Figure 5. Diet and inflammation affect the infant gut microbiota.
Supplementary Figure 6. Potential sources of infant-colonizing microbes.
Supplementary Figure 7. Infant oral microbiotas assemble according to neutral rules.
Supplementary Figure 8. Patterns of selection and neutral assembly are consistent across ontogeny and geography.
Supplementary Figure 9. Neutral processes explain microbial community assembly across age groups and geography.
Supplementary Figure 10. Gut microbiota structure among Tsimane villages.
Supplementary Figure 11. Bacterial taxa discriminate between village types.

Supplementary Table 1. Demographics of Tsimane villages and study subjects.

Supplementary Figure 1

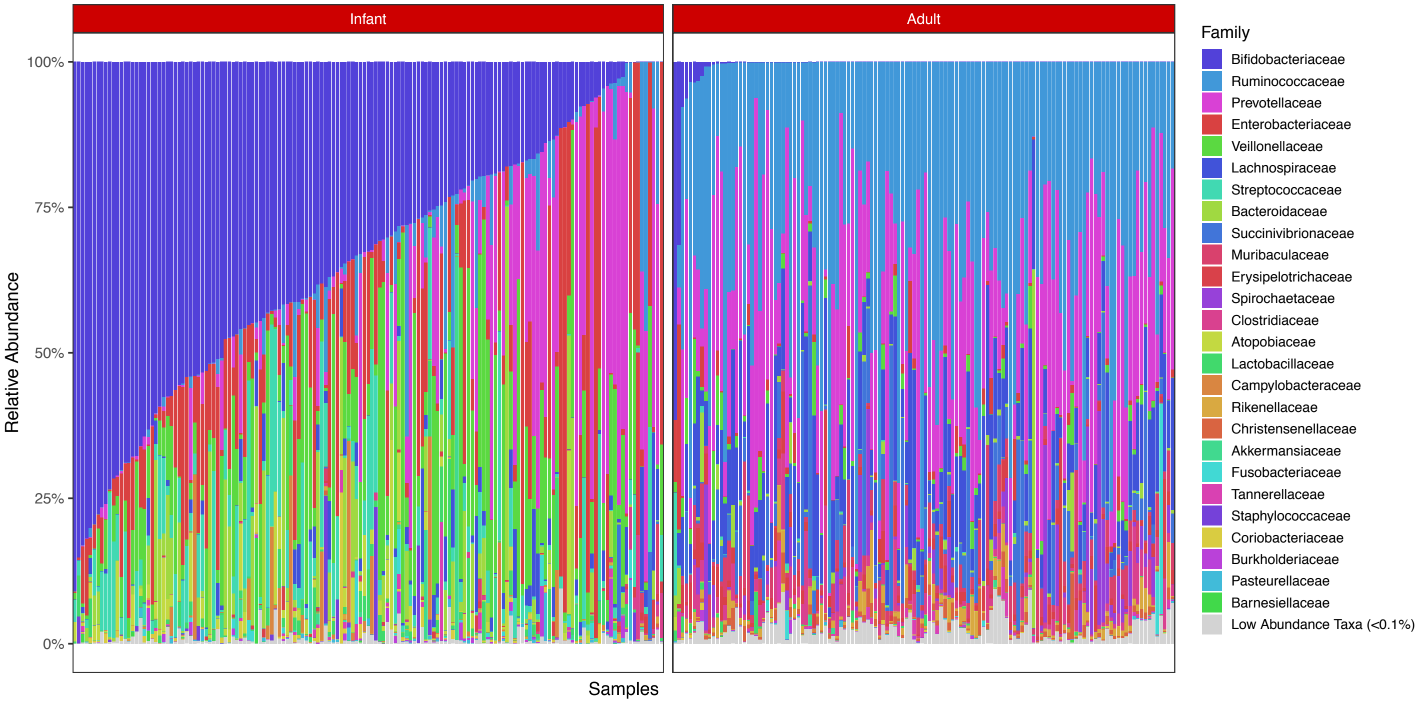

Supplementary Figure 1. **Dominant bacterial families of Tsimane stool microbiotas.** Stacked bar chart of the relative abundance of each bacterial family in each sample. Samples are sorted along the x-axis by their relative abundance of *Bifidobacteriaceae*, the most abundant family. Families that do not make up at least 0.1% of any sample are shown in gray.

Supplementary Figure 2

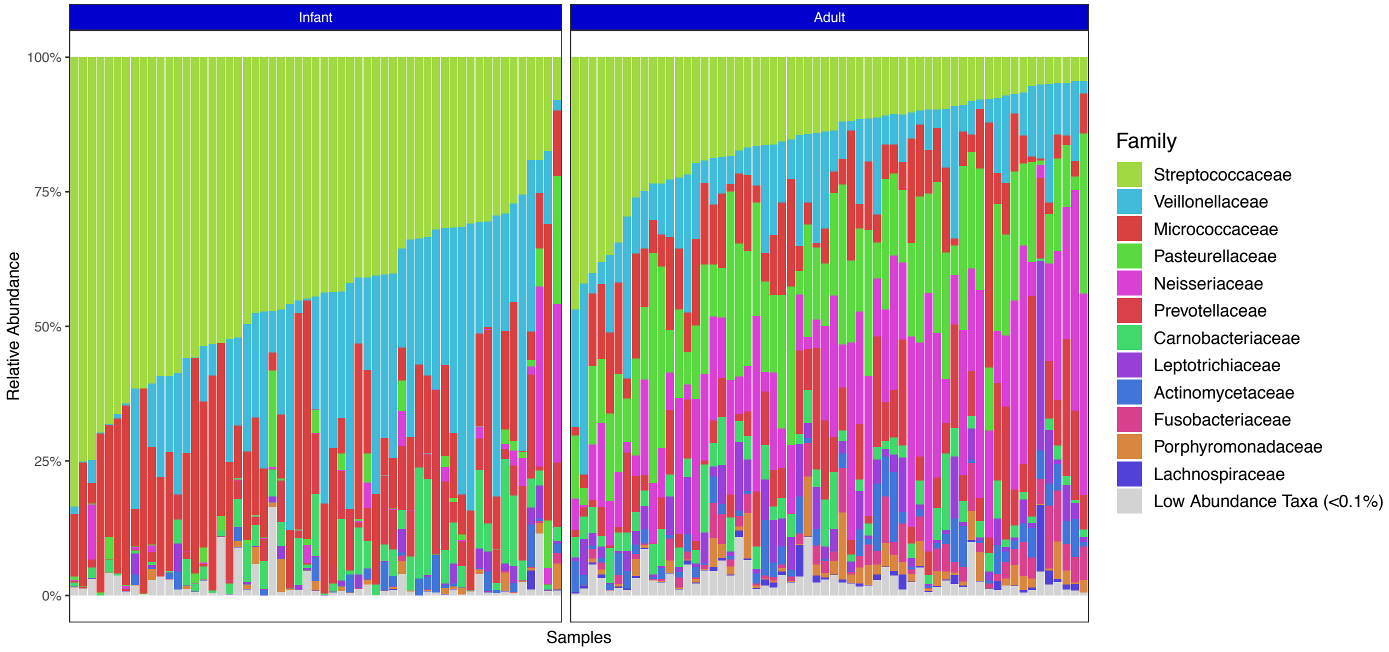

Supplementary Figure 2. **Dominant bacterial families of Tsimane oral microbiotas.** Stacked bar chart of the relative abundance of each bacterial family in each sample. Samples are sorted along the x-axis by their relative abundance of *Streptococcaceae*, the most abundant family. Families that do not make up at least 0.1% of any sample are shown in gray.

Supplementary Figure 3

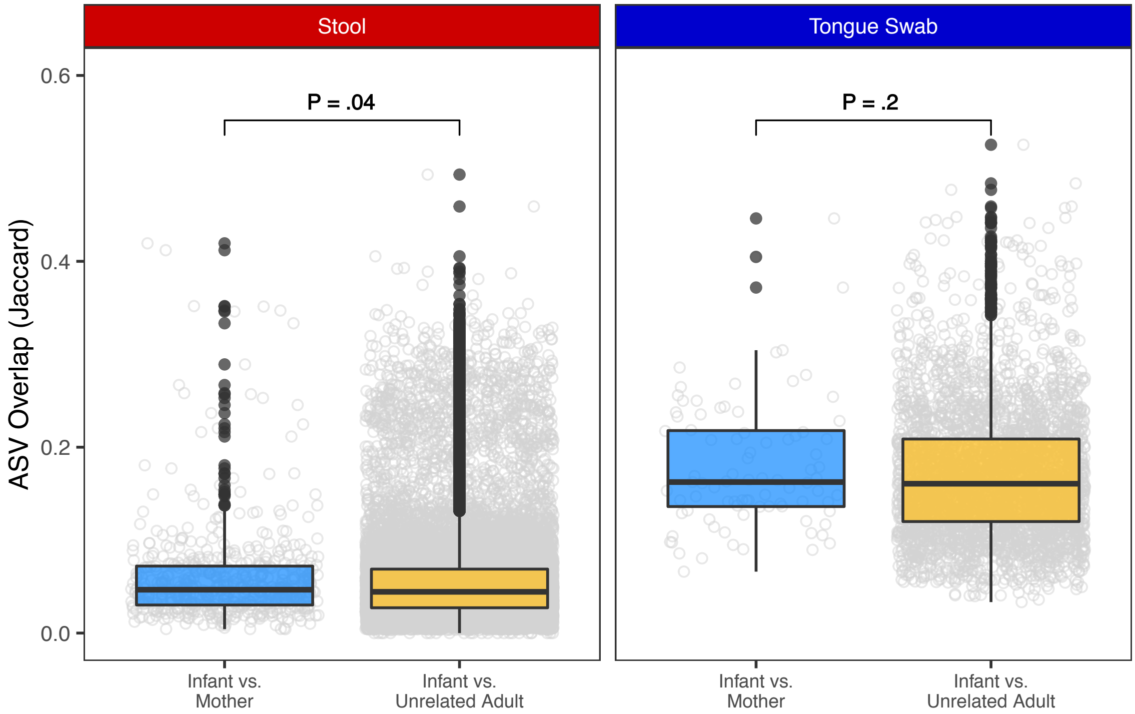

Supplementary Figure 3. **Gut and oral microbiota sharing between infants and mothers or unrelated adults.** Average number of ASVs shared between infants and their mother or infants and unrelated adults in stool and tongue swab microbiotas. (Wilcoxon Rank Sum Test, two-sided, stool: adjusted P = .04, tongue swabs: adjusted P = .2). n comparisons per group: Infant Stool vs. Mother Stool: 530; Infant Stool vs. Unrelated Adult Stool: 19,360; Infant Tongue Swab vs. Mother Tongue Swab: 87; Infant Tongue Swab vs. Unrelated Adult Tongue Swab: 3,333. The center denotes the median, the lower and upper hinges correspond to the first and third quartiles, respectively, while the upper whisker extends to the largest value no further than 1.5 * inter-quartile range from the hinge and the lower whisker extends from the hinge to the smallest value at most 1.5 * inter-quartile range of the hinge.

Supplementary Figure 4

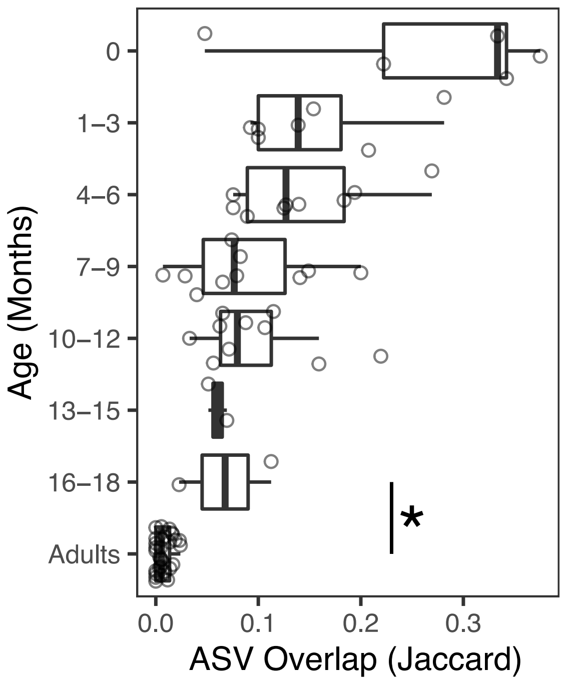

Supplementary Figure 4. **Gut and oral microbiotas diverge as infants age.** Average number of ASVs shared between body sites within a subject based on samples collected within 14 days of each other. Boxplot color darkens with increased age. Adults and 16-18 month olds were the only sequential age groups whose microbiotas were significantly different from one another (Wilcoxon Rank Sum Test, * adjusted P = .03). n comparisons per age group: 0 months: 5, 1 - 3 months 7, 4 - 6 months 9, 7 - 9 months 10, 10 - 12 months 10, 13 - 15 months 2, 16 - 18 months 2, Adult 30. The center denotes the median, the lower and upper hinges correspond to the first and third quartiles, respectively, while the upper whisker extends to the largest value no further than 1.5 * inter-quartile range from the hinge and the lower whisker extends from the hinge to the smallest value at most 1.5 * inter-quartile range of the hinge.

Supplementary Figure 5

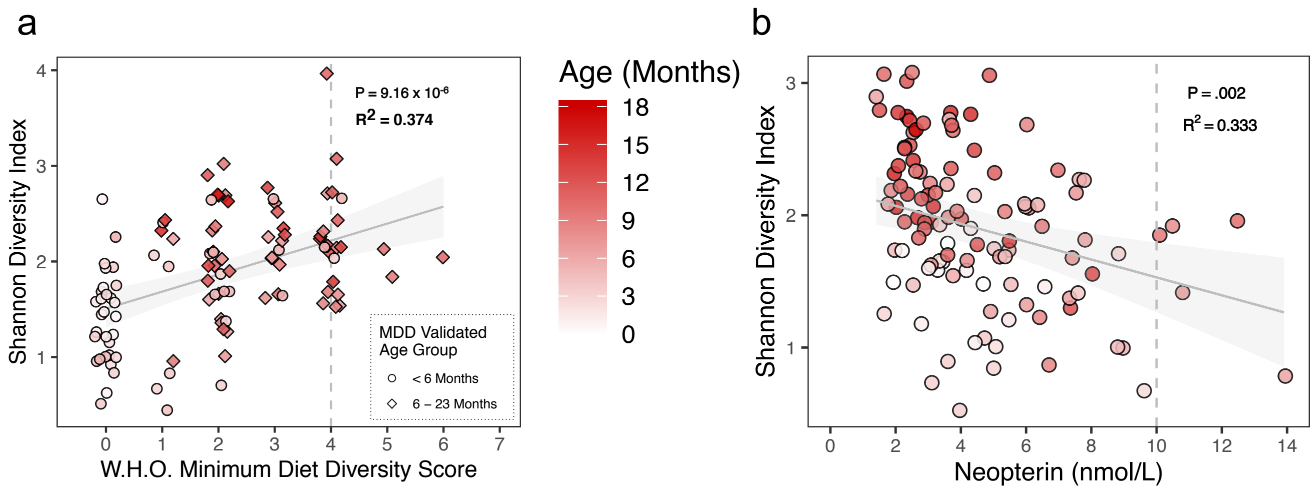

Supplementary Figure 5. **Diet and inflammation affect the infant gut microbiota.** (a) The World Health Organization's Minimum Diet Diversity score regressed against the Shannon diversity index of the infant stool samples. The MDD was validated to assess the diversity of complementary foods of 6-23 month old children in a diverse range of cultural contexts. It should only be interpreted as an estimate of micronutrient sufficiency in children aged 6-23 months, which are denoted by a diamond shape, and the color darkens as the subject's age increases. MDD of 4 is considered to be sufficiently diverse, as indicated by the vertical dashed line. Lines indicate the linear mixed-effects regression of diversity on MDD, while treating subject as a random effect. The shading indicates the 95% confidence interval. The conditional R^2 describes the proportion of variation explained by both fixed and random factors. (B) Levels of fecal neopterin, an inflammatory marker normally produced by activated macrophages, regressed against the Shannon diversity index in infant stool samples. Lines indicate the linear mixed-effects regression of diversity on neopterin, while treating subject as a random effect. The shading indicates the 95% confidence interval. The conditional R^2 describes the proportion of variation explained by both fixed and random factors, and was calculated using the R package, 'piecewiseSEM'. A neopterin level above 10, as indicated by the vertical dashed line, is considered to indicate clinically relevant inflammation.

Supplementary Figure 6

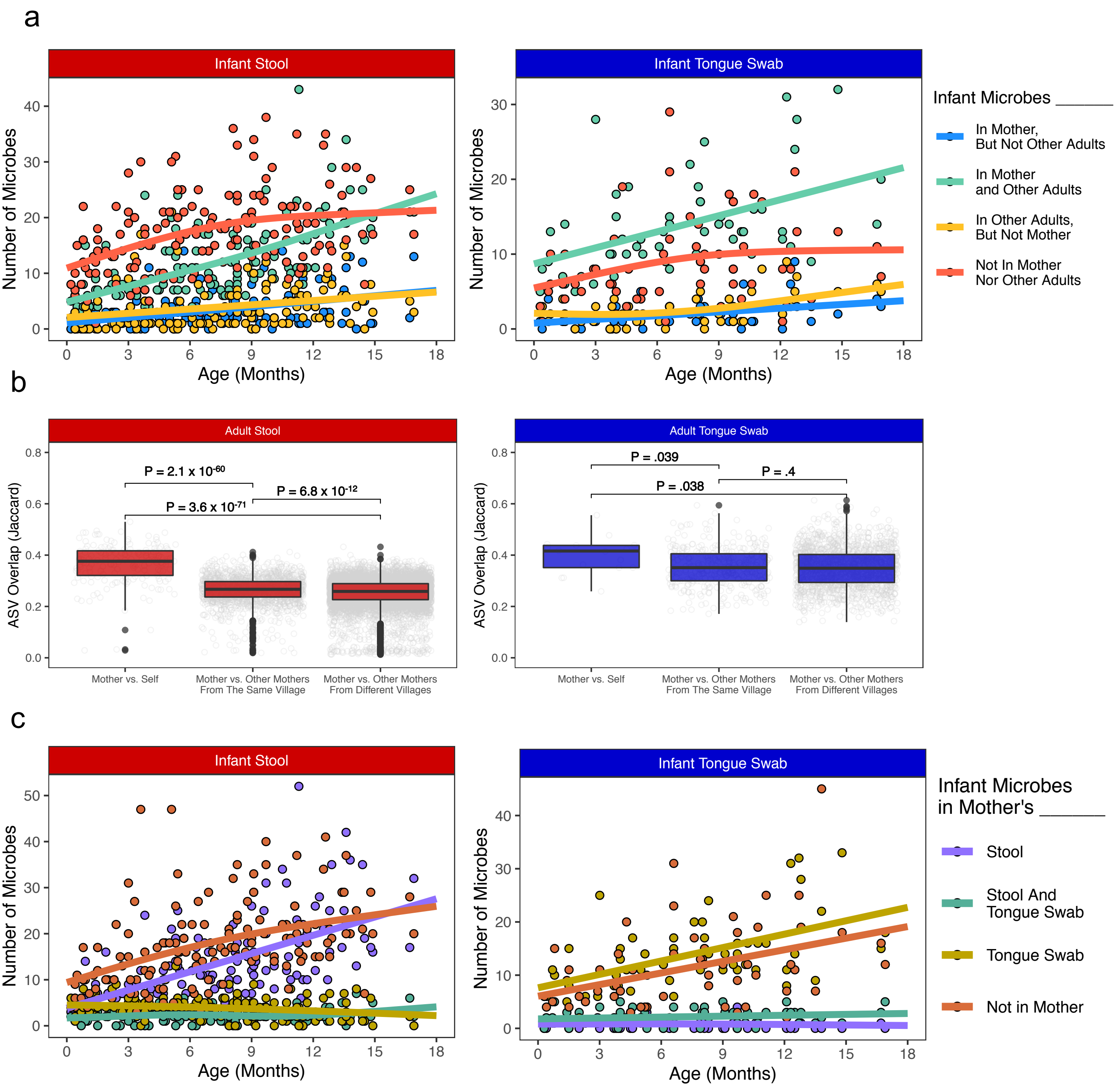

Supplementary Figure 6. **Potential sources of infant-colonizing microbes.** (a) Scatter plot of the average number of ASVs found in each infant stool sample that were also found in that infant's mother but not in other adults from their village (blue), in both their mother and other mothers from their village (green), in other mothers from the infant's village but not their mother (yellow), or neither in their mother or other adults (orange). Non-parent adults from each village were down-sampled to control for differences in the number of samples collected from each village. Left panel – infant stool samples, right panel – infant tongue swabs. (b) Average number of ASVs shared between multiple samples from the same mother, between mothers living in the same village, or mothers living in different villages. Wilcoxon Rank Sum Test, two-sided. P-values were adjusted using Holm's method. Left panel – adult stool samples, right panel – adult tongue swabs. n comparisons per group: Mother vs. Self - Adult Stool 171, Tongue Swab 16; Mother vs. Other Mothers From The Same Village - Adult Stool 1573, Tongue Swab 334; Mother vs. Other Mothers From Different Villages - Adult Stool 7034, Tongue Swab 1541. The center denotes the median, the lower and upper hinges correspond to the first and third quartiles, respectively, while the upper whisker extends to the largest value no further than 1.5 * inter-quartile range from the hinge and the lower whisker extends from the hinge to the smallest value at most 1.5 * inter-quartile range of the hinge. (c) Scatter plot of the average number of ASVs in each infant stool sample that was also found in their mother's stool but not in her tongue swab (purple), in both their mother's stool and tongue swab (green), in their mother's tongue swab but not their mother's stool (yellow), or not found in any of their mother's samples (orange).

Supplementary Figure 7

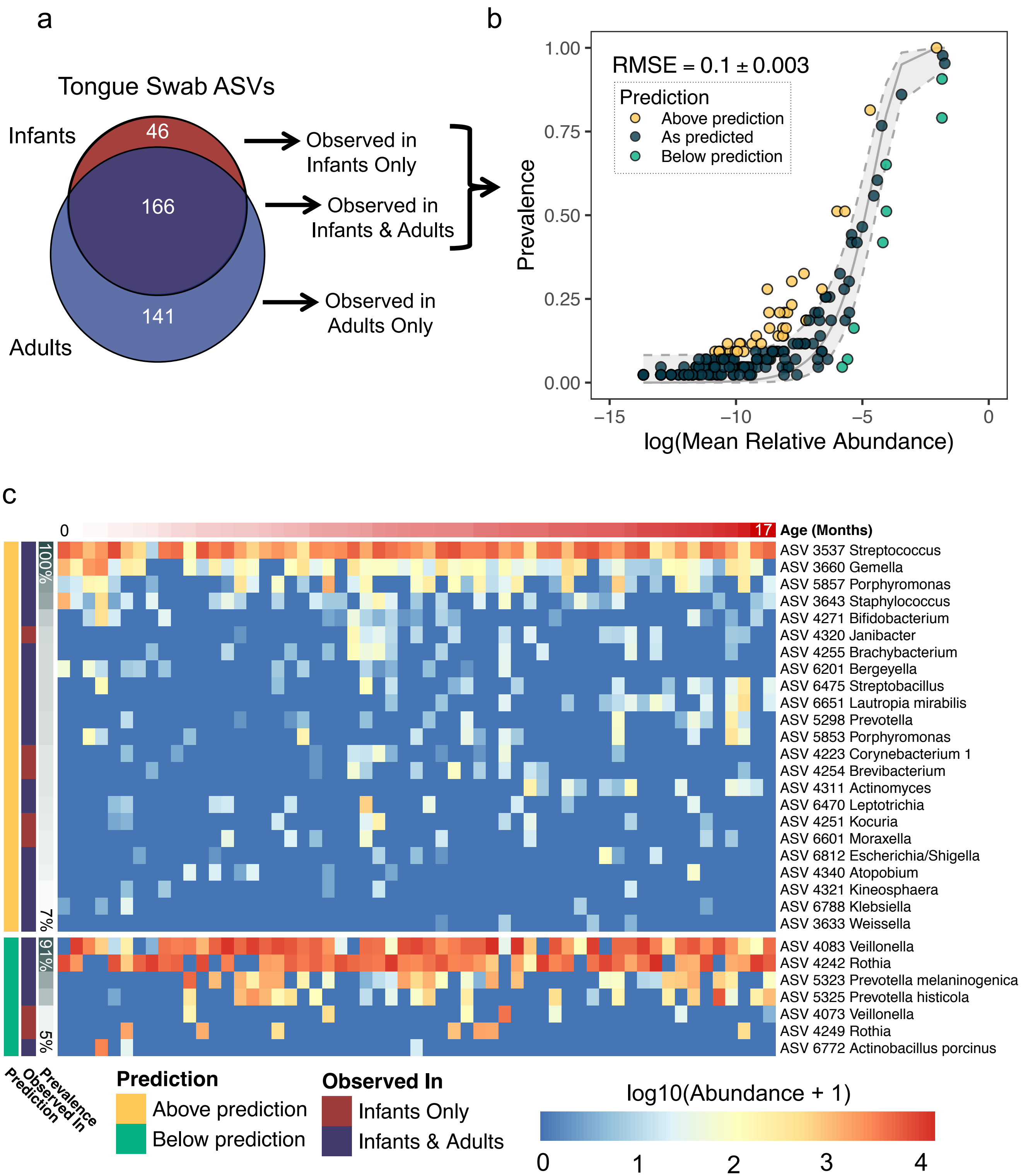

Supplementary Figure 7. **Infant oral microbiotas assemble according to neutral rules.** (a) Venn diagram showing the number of ASVs observed in infant and adult tongue swabs. Only taxa found in infant tongue swabs were included in part b. (b) Neutral community model (NCM) fit to each ASV observed in the tongue swabs of Tsimane infants. Points are colored according to whether the taxon prevalence in infant samples is above (yellow), at (blue), or below (green) the predicted prevalence according to the NCM. Average RMSE (± standard deviation) was calculated from 1,000 bootstrap resamplings. (c) Heatmap of the $\log_{10}$ normalized abundances of the ASVs from infant tongue swabs that were observed either to be consistently above (yellow) or below (green) their predicted prevalence in part b. Rows are sorted by taxa prevalence, columns are sorted by the subject's age (months).

Supplementary Figure 8

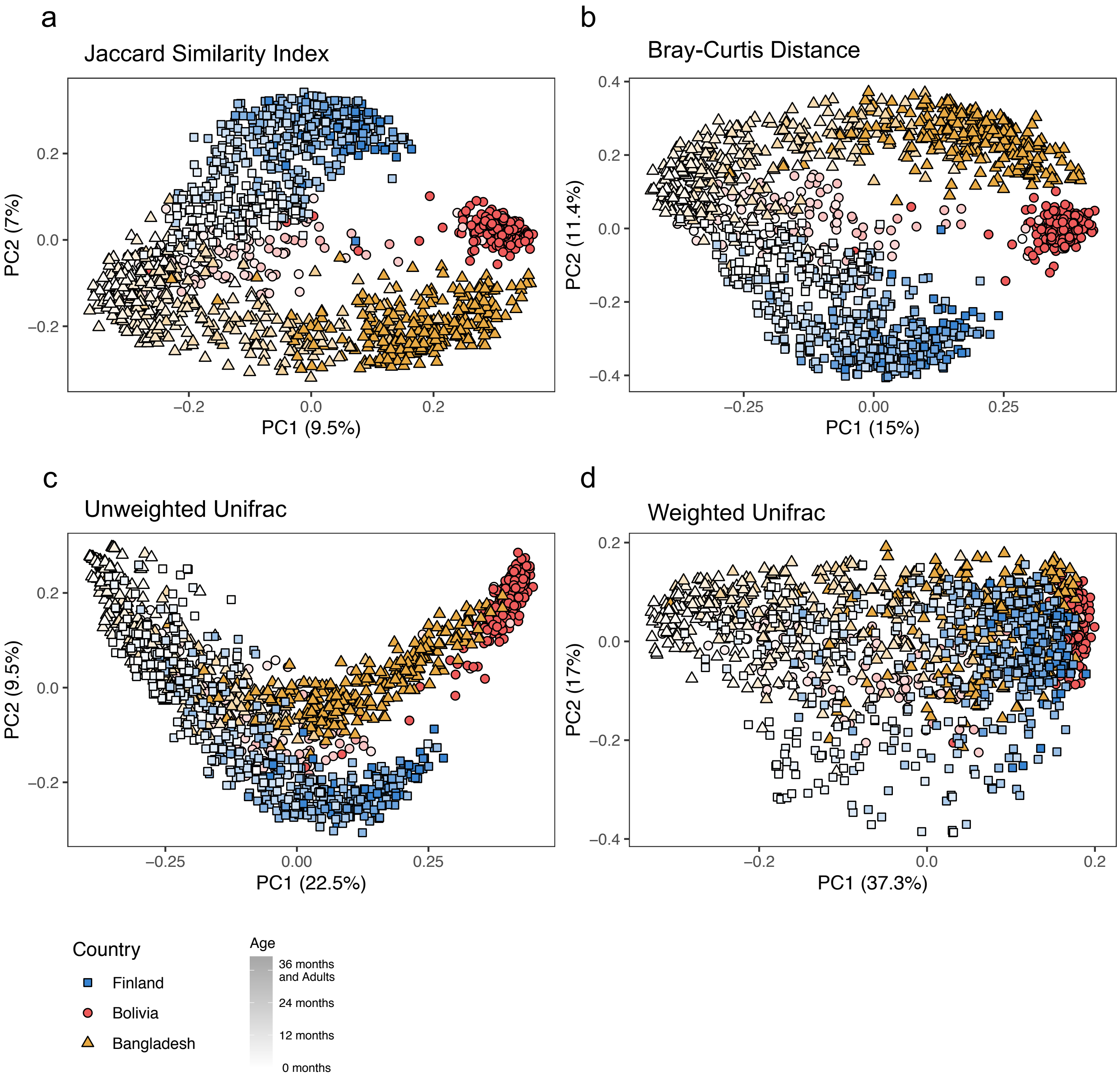

Supplementary Figure 8. **Patterns of selection and neutral assembly are consistent across ontogeny and geography.** (a-d) Principal coordinate analysis (PCoA) on the (a) Jaccard similarity index, (b) Bray-Curtis distance, (c) unweighted Unifrac distance, and (d) weighted Unifrac distance of 16S rRNA profiles from stool samples from Bolivian (Tsimane) dyads, as well as previously published 16S rRNA datasets of Bangladeshi and Finnish subjects. Points are shaped and colored according to country, and the color darkens with increased age of the subject (Finland - blue squares, Bolivia (Tsimane) – red circles, Bangladesh – yellow triangles).

Supplementary Figure 9

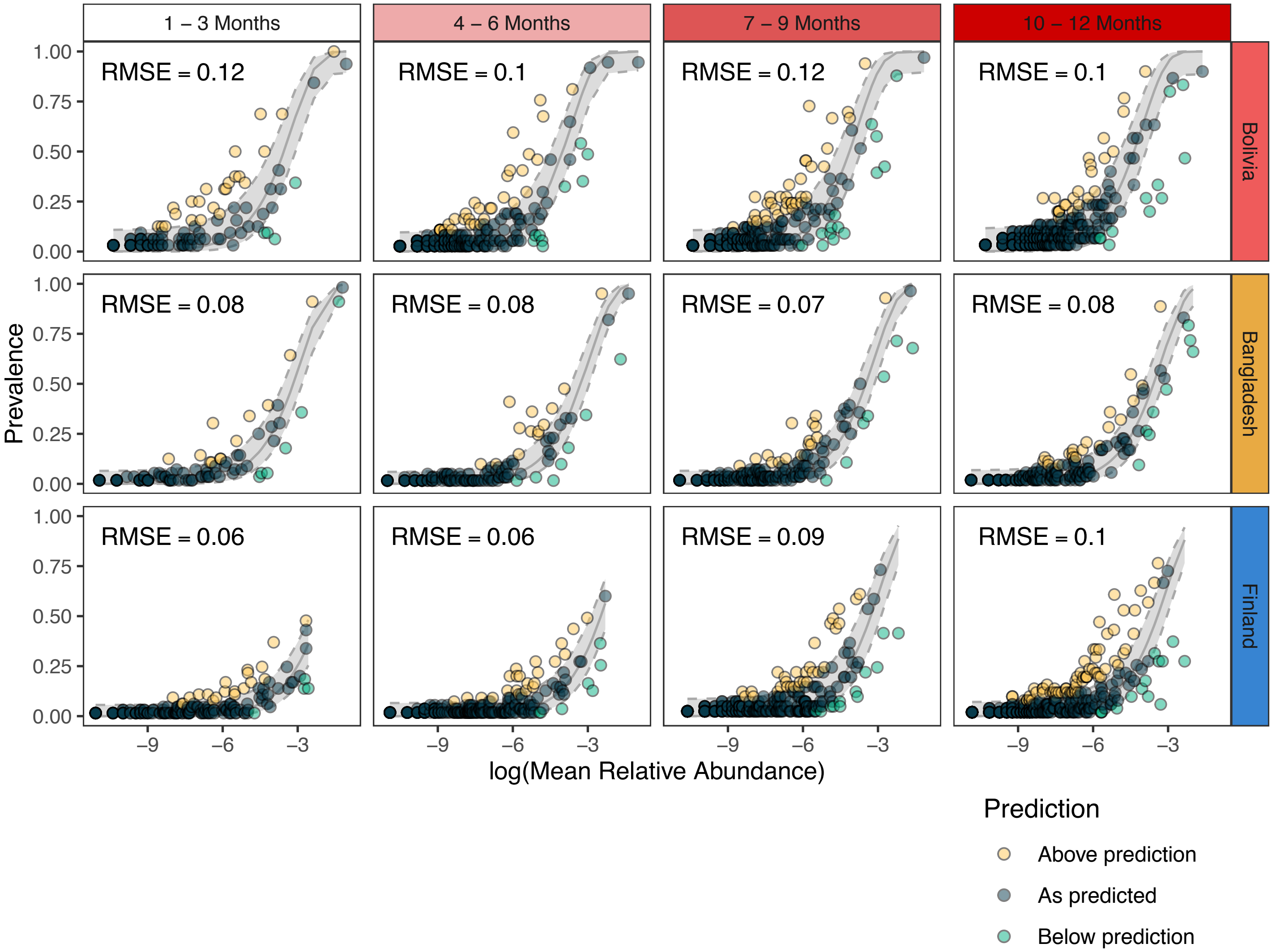

Supplementary Figure 9. **Neutral processes explain microbial community assembly across age groups and geography.** Scatterplot of the prevalence of each ASV in the infant gut versus its mean relative abundance in the regional species pool for each age group and country. The gray line is their predicted distribution (shaded area is 95% confidence interval) based on the neutral community model. Points are colored by the ASV's fit to the model: above prediction – yellow, at prediction – blue, below prediction – green.

Supplementary Figure 10

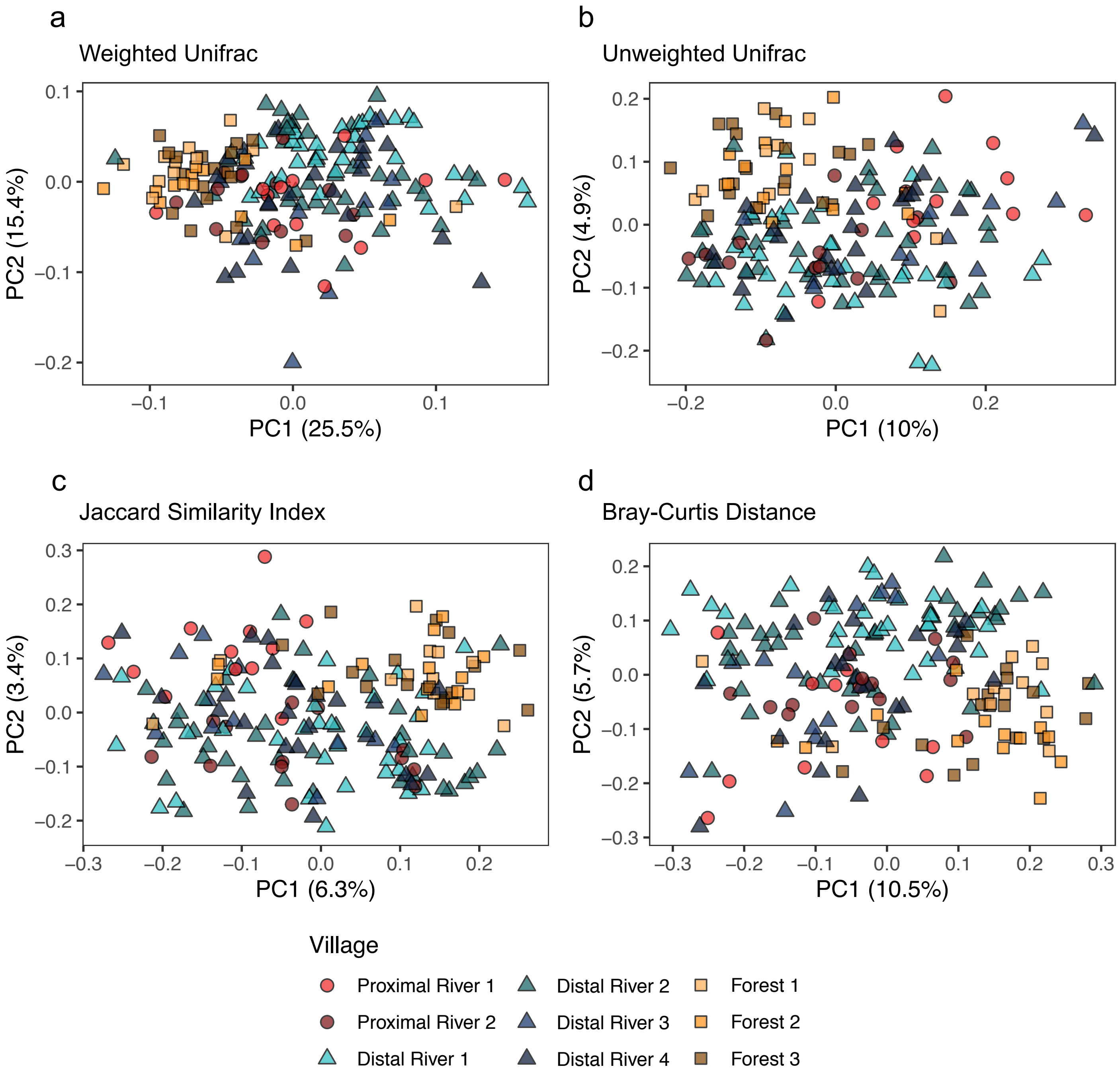

Supplementary Figure 10. **Gut microbiota structure among Tsimane villages**. Principal coordinate analysis (PCoA) on the (a) weighted Unifrac distance, (b) unweighted Unifrac distance, (c) Jaccard similarity index, and (d) Bray-Curtis distance of 16S rRNA profiles from adult stool samples. Village types (‘proximal’ river, ‘distal’ river, and ‘forest’) have significantly different distributions (PERMANOVA with 1000 permutations, P = .001). Samples are colored by village and their shapes denote the village type (proximal river villages – circles, distal river villages – triangles, forest villages – squares).

Supplementary Figure 11

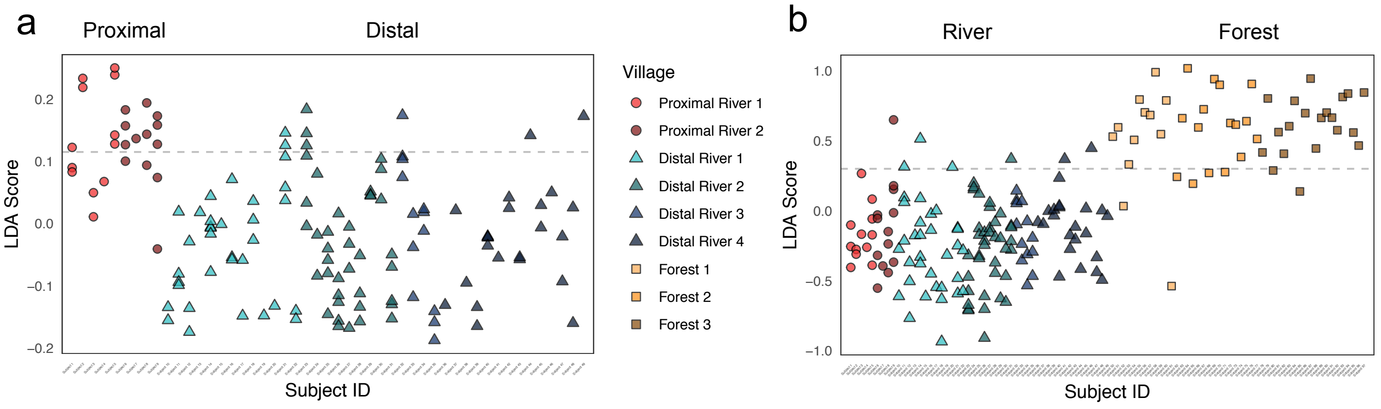

Supplementary Figure 11. **Bacterial taxa discriminate between village types.** (a) The LDA score of each adult stool sample was calculated from a phylogeny-based form of linear discriminant analysis. Higher scores indicate samples more likely to have been collected from a proximal river village, while lower scores indicate samples more likely to have been collected from a distal river village. The dashed gray line represents the optimal LDA score cut-off between groups, where it accurately classifies 87.3% of samples. Samples are colored by village, and their shapes represent the village type (proximal river – circles, distal river – triangles, forest – squares). (b) The LDA score of each adult stool sample was calculated from a phylogeny-based form of linear discriminant analysis. Higher scores indicate samples more likely from a forest village, while lower scores indicate samples more likely from a river village. The dashed gray line represents the optimal LDA score cut-off between groups, where it accurately classifies 91.8% of samples. The colors and shapes are the same as in Panel a.

Supplementary Table 1

Village	Age Class	Number of Subjects	Stool Samples	Tongue Swabs	All Samples	Distance from City (Km)	Population*
Proximal River 1	*Adult*	6	12	8	47	14.3	331
	Child	0	0	0			
	Infant	6	20	7			
Proximal River 2	*Adult*	4	13	6	42	18.5	292
	Child	0	0	0			
	Infant	4	17	6			
Distal River 1	*Adult*	13	34	13	92	29.8	151
	Child	0	0	0			
	Infant	11	33	12			
Distal River 2	*Adult*	9	39	18	128	33.5	253
	Child	0	0	0			
	Infant	10	53	18			
Distal River 3	*Adult*	5	13	5	36	37.5	101
	Child	0	0	0			
	Infant	5	14	4			
Distal River 4	*Adult*	14	23	12	65	42.5	206
	Child	0	0	0			
	Infant	12	19	11			
Forest 1	*Adult*	12	12	0	21	31	61
	Child	8	8	0			
	Infant	1	1	0			
Forest 2	*Adult*	16	16	0	27	61	114
	Child	11	11	0			
	Infant	0	0	0			
Forest 3	*Adult*	20	20	0	25	68	101
	Child	5	5	0			
	Infant	0	0	0			

Supplementary Table 1**. Demographics of Tsimane villages and study subjects.** Villages are listed in order of increasing distance from the closest market town. Age classes are defined as Infant – under 2 years, Child – 2 to 13 years old, Adult – 14 years old and older. The 73 fecal samples collected from forest communities were collected in July 2009, while the remaining fecal and oral samples from proximal and distal river communities were collected between September 2012 and March 2013. *Population sizes for forest villages were determined during a 2009 census, while the remaining were determined in a 2012 census.
