# Supplementary material for: Microbiota assembly, structure, and dynamics among Tsimane horticulturalists of the Bolivian Amazon
Source: Nat Commun. 2020 Jul 29;11:3772. doi: 10.1038/s41467-020-17541-6 (PMC7391733; doi:10.1038/s41467-020-17541-6)
Supplement: Supplementary file 3 — Reporting Summary [file 41467_2020_17541_MOESM3_ESM.pdf]

nature research

Corresponding author(s): David Relman

Last updated by author(s): June 22, 2020

Reporting Summary

Nature Research wishes to improve the reproducibility of the work that we publish. This form provides structure for consistency and transparency in reporting. For further information on Nature Research policies, see Authors & Referees and the Editorial Policy Checklist .

Statistics

For all statistical analyses, confirm that the following items are present in the figure legend, table legend, main text, or Methods section.

n/a	Confirmed	
☐	☒	The exact sample size (*n*) for each experimental group/condition, given as a discrete number and unit of measurement
☒	☐	A statement on whether measurements were taken from distinct samples or whether the same sample was measured repeatedly
☐	☒	The statistical test(s) used AND whether they are one- or two-sided<br>*Only common tests should be described solely by name; describe more complex techniques in the Methods section.*
☐	☒	A description of all covariates tested
☒	☐	A description of any assumptions or corrections, such as tests of normality and adjustment for multiple comparisons
☐	☒	A full description of the statistical parameters including central tendency (e.g. means) or other basic estimates (e.g. regression coefficient) AND variation (e.g. standard deviation) or associated estimates of uncertainty (e.g. confidence intervals)
☐	☒	For null hypothesis testing, the test statistic (e.g. *F*, *t*, *r*) with confidence intervals, effect sizes, degrees of freedom and *P* value noted<br>*Give P values as exact values whenever suitable.*
☒	☐	For Bayesian analysis, information on the choice of priors and Markov chain Monte Carlo settings
☒	☐	For hierarchical and complex designs, identification of the appropriate level for tests and full reporting of outcomes
☒	☐	Estimates of effect sizes (e.g. Cohen's *d*, Pearson's *r*), indicating how they were calculated

Our web collection on statistics for biologists contains articles on many of the points above.

Software and code

Policy information about availability of computer code

Data collection	No special software was used during data collection.
Data analysis	All data analysis was performed in R (verion 3.6.0). R packages used include: dada2 (Version 1.14.0), phyloseq (version 1.28.0), ggplot2 (version 3.2.0), pheatmap (version 1.0.12), treeDA (version 0.0.3), ggpubr (version 0.2.1) , vegan (version 2.5.5), igraph (version 1.2.4.1), UniFrac (version 1.1), SpiecEasi (version 1.0.6), minpack.lm (version 1.2-1), tyRa (version 0.1.0, available at https://danielsprockett.github.io/tyRa/). Analysis code and data is available at https://purl.stanford.edu/tv993xn7633. QIIME2 (version 2019.1) was used to demultiplex samples and for placing sequence variants into a phylogenetic tree using the fragment insertion function.

For manuscripts utilizing custom algorithms or software that are central to the research but not yet described in published literature, software must be made available to editors/reviewers. We strongly encourage code deposition in a community repository (e.g. GitHub). See the Nature Research guidelines for submitting code & software for further information.

Data

Policy information about availability of data

All manuscripts must include a data availability statement. This statement should provide the following information, where applicable:

- Accession codes, unique identifiers, or web links for publicly available datasets
- A list of figures that have associated raw data
- A description of any restrictions on data availability

The dataset generated during this current study is available at NCBI Sequence Read Archive (BioProject ID PRJNA574920 [https://www.ncbi.nlm.nih.gov/bioproject/PRJNA574920]). Additional datasets analyzed in this study are available at European Nucleotide Archive (Accession code PRJEB5482 [https://www.ebi.ac.uk/ena/data/view/PRJEB5482])26 and NCBI Sequence Read Archive (BioProject ID PRJNA290380 [https://www.ncbi.nlm.nih.gov/bioproject/PRJNA290380/])27. The SILVA nr database v132 is available on the DADA2 github repository (https://benjjneb.github.io/dada2) or www.arb-silva.de. The source data underlying all figures are provided as a Source Data file.

Field-specific reporting

Please select the one below that is the best fit for your research. If you are not sure, read the appropriate sections before making your selection.

☐ Life sciences ☐ Behavioural & social sciences ☒ Ecological, evolutionary & environmental sciences

For a reference copy of the document with all sections, see nature.com/documents/nr-reporting-summary-flat.pdf

Ecological, evolutionary & environmental sciences study design

All studies must disclose on these points even when the disclosure is negative.

Study description

Microbial communities were profiled in stool and tongue swabs of collected from mother-infant dyads living an indigenous lifestyle in the Bolivian Amazon. Statistical modeling was then performed on these profiles to assess the patterns of microbial transmission between mothers and children, as well as the contribution of neutral processes. These samples were then compared to previously published infant and adult datasets from Finnish and Bangladeshi populations.

Research sample

Human-associated microbial communities were profiled using 16S rRNA gene amplicon sequencing of stool and oral swabs from 52 Tsimane families living in six villages located along the Maniqui River in the Bolivian lowlands of the Amazon basin. One set of samples were collected in 2012 – 2013 from 48 infants (0-2 years of age) and 51 mothers (14 or more years of age). Samples from unpaired dyads (infants when their mother was not enrolled, or mothers with no infant enrolled), were removed from further analysis. In addition, stool samples collected from 73 Tsimane individuals in 2009 were also profiled. Previously published datasets from infants and adults were also analyzed (see above).

Biospecimens collected in 2012-2013 were collected from families participating in a longitudinal study on infant feeding transitions, conducted by co-author Martin. For this study, all families with infants < 1 year of age that could be located were recruited from six study villages. These villages represented all villages in the upriver region of Tsimane territories. They were chosen because they are in close proximity to one another (logistically necessary for the investigator to conduct repeat observations with participant families in the 8-month study period), but also vary in access to a large market town. Stool samples collected in 2009 were collected by Martin as a pilot study to examine Tsimane microbial composition, in collaboration with co-author Relman. Samples were collected in one month from three villages from all consenting participants seen by the Tsimane Health and Life History Project medical team, in relation to a multi-year investigation on health and aging. At the time, the THLHP mobile team travelled year-round to Tsimane villages providing primary medical care and collecting biodemographic data from the following participants in each visited village: all adults over 40 y, all children under 5 y, a random sample of people age 5-39 y, and any other village residents desiring medical attention.

Sampling strategy

All Tsimane mothers with children under 2 years of age that were living in the sampled villages were approached for study enrollment.

Data collection

Author Melanie Martin collected samples from participants during the 2009 and 2012-2013 field seasons. Data on breastfeeding status, 24-hour dietary recall, anthropometrics, and symptoms of infectious disease was also collected via a oral interview as part of a separate study of changes in infant feeding and related maternal and infant health outcomes (Martin et al. 2016, Soc. Sci. Med.).

Timing and spatial scale

Samples were collected from 6 riverine Tsimane villages and 3 forest villages. The samples from the riverine villages were collected from September 2012 through March 2013, while samples from forest villages were collected in July 2009. Biospecimen for this study were collected opportunistically during separate investigations carried out by Tsimane Health and Life History Project researchers in 2009 and 2012-2013.

Data exclusions

A small number of samples failed PCR amplification for unknown reasons, resulting in a very low number of reads (<1,000). These samples were removed from further analysis. Samples from unpaired dyads (infants where the mother was not enrolled, or mothers where their children were not enrolled), were also removed.

Reproducibility

This study mainly involved statistical modeling of observational data, so there were no experiments to reproduce.

Randomization

Subjects were not randomized because this study was mainly observational, and did not involve subject groups.

Blinding

Blinding was not relevant to this study because it did not involve a treatment group.

Did the study involve field work? ☒ Yes ☐ No

Field work, collection and transport

Field conditions

The field conditions were 9 Tsimane villages over two different field seasons (2009 and 2012-2013). All villages are located within the Tsimane territory in the lowland Amazonian region, in the Beni Department of Bolivia. Six villages were located along the Maniqui River, while the other 3 were located in interior forest along an old logging road. Temperatures in the region average 24-26°C, with minimum and maximum average monthly temperatures of 18-36°C. Average total annual rainfall is 1764 mm (http://www.senamhi.gob.bo/sismet/). However, rainfall is seasonal, with precipitation highest during the months of November – March (average total precipitation/month 242 mm) and lowest during the months of June – August (average total

	precipitation/month 52 mm)
Location	Nine Tsimane villages in the Bolivian Amazon basin. Villages were located within 10 – 70 km of the town of San Borja (14.8585° S, 66.7470° W). The small population size of the villages prevent us from disclosing their exact locations, in order to retain anonymity.
Access and import/export	All study protocols were approved by the University of California Santa Barbara Institutional Review Board on Human Subjects (IRB Protocols # ANTH-GU-MI-010-3U, submission ID 09-312, approved 8/21/2009; ANTH-GU-MI-010-19N, submission ID 12-354 approved 6/08/2012). Permission to conduct research was granted to the Tsimane Life History Project (THLHP) and their research affiliates. The THLHP maintains formal agreements with the local municipal government of San Borja and the Tsimane governing body (Gran Consejo Tsimane). Participants provided verbal consent for themselves and infants, as most Tsimane women are illiterate.
Disturbance	No disturbances.

Reporting for specific materials, systems and methods

We require information from authors about some types of materials, experimental systems and methods used in many studies. Here, indicate whether each material, system or method listed is relevant to your study. If you are not sure if a list item applies to your research, read the appropriate section before selecting a response.

Materials & experimental systems

n/a	Involved in the study
☒	☐ Antibodies
☒	☐ Eukaryotic cell lines
☒	☐ Palaeontology
☒	☐ Animals and other organisms
☐	☒ Human research participants
☒	☐ Clinical data

Methods

n/a	Involved in the study
☒	☐ ChIP-seq
☒	☐ Flow cytometry
☒	☐ MRI-based neuroimaging

Human research participants

Policy information about studies involving human research participants

Population characteristics	This study included data collected from mothers (aged 14-44) and infants (aged 0-2 years) and a sample of mixed-age and gender participants (children and adults aged 1-78), all with self-identified Tsimane ethnicity. The Tsimane people are an indigenous forager-horticulturalist population inhabiting the Bolivian Amazon basin (pop. ~16,000) between the eastern slopes of the Andes mountains and the llanos de Moxos. There are approximately 95 Tsimane villages in the territory, ranging in size from 40-550 individuals. Tsimane language is an isolate, unrelated to dominant languages of Spanish, Quechua and Aymara, but close to Moseten language. Analyses of mtDNA from the first hypervariable control region have shown distinct haplotypes from Europeans, and relatively low admixture with other indigenous populations. Average life expectancy is 53 years. Other details of the population characteristics are available in the manuscript.
Recruitment	As described above, Tsimane populations are small, and therefore every mother with children under 2 years of age was approached to be enrolled in this study.
Ethics oversight	All study protocols were approved by the University of California Santa Barbara Institutional Review Board on Human Subjects (IRB Protocols # ANTH-GU-MI-010-3U, submission ID 09-312, approved 8/21/2009; ANTH-GU-MI-010-19N, submission ID 12-354 approved 6/08/2012). Permission to conduct research was granted to the Tsimane Life History Project (THLHP) and their research affiliates. Consent was obtained from village leaders and community members during initial meetings upon starting research activities in each village. Consent was then obtained verbally from individual participants prior to all data collection. Mothers provided parental consent to collect samples from infants.

Note that full information on the approval of the study protocol must also be provided in the manuscript.
