# Supplementary material for: Microbiota assembly, structure, and dynamics among Tsimane horticulturalists of the Bolivian Amazon
Source: Nat Commun. 2020 Jul 29;11:3772. doi: 10.1038/s41467-020-17541-6 (PMC7391733; doi:10.1038/s41467-020-17541-6)
Supplement: Supplementary file 4 — Description of Additional Supplementary Files [file 41467_2020_17541_MOESM4_ESM.pdf]

Description of Additional Supplementary Files

File Name: Supplementary Data 1
Description: Detailed sample data. This table includes the per-sample metadata for sample type, subject age, gender, family structure, geography, diet, and neopterin levels
